# *Lactobacillus plantarum* possesses the capability for wall teichoic acid backbone alditol switching

**DOI:** 10.1186/1475-2859-11-123

**Published:** 2012-09-11

**Authors:** Peter A Bron, Satoru Tomita, Iris I van Swam, Daniela M Remus, Marjolein Meijerink, Michiel Wels, Sanae Okada, Jerry M Wells, Michiel Kleerebezem

**Affiliations:** 1 TI Food & Nutrition, Nieuwe Kanaal 9A, 6709 PA, Wageningen, The Netherlands; 2 NIZO Food Research, Kernhemseweg 2, 6718ZB, Ede, The Netherlands; 3 Kluyver Centre for Genomics of Industrial Fermentation, Julianalaan 67, 2628 BC, Delft, The Netherlands; 4 Department of Applied Biology and Chemistry, Faculty of Applied Bio-Science, Tokyo University of Agriculture, 1-1-1 Sakuragaoka, Setagaya-ku, Tokyo, 156-8502, Japan; 5 Host-Microbe Interactomics, Wageningen University, Marijkeweg 40, 6709 PG, Wageningen, The Netherlands; 6 Netherlands Consortium for Systems Biology, Science Park 904, 1098 XH, Amsterdam, The Netherlands; 7 Laboratory for Microbiology, Wageningen University, Dreijenplein 10, 6703 HB, Wageningen, The Netherlands

**Keywords:** *Lactobacillus plantarum*, Probiotic, Wall teichoic acid, Lipoteichoic acid, *tag* and *tar* genes, Immunomodulation

## Abstract

**Background:**

Specific strains of *Lactobacillus plantarum* are marketed as health-promoting probiotics. The role and interplay of cell-wall compounds like wall- and lipo-teichoic acids (WTA and LTA) in bacterial physiology and probiotic-host interactions remain obscure. *L. plantarum* WCFS1 harbors the genetic potential to switch WTA backbone alditol, providing an opportunity to study the impact of WTA backbone modifications in an isogenic background.

**Results:**

Through genome mining and mutagenesis we constructed derivatives that synthesize alternative WTA variants. The mutants were shown to completely lack WTA, or produce WTA and LTA that lack D-Ala substitution, or ribitol-backbone WTA instead of the wild-type glycerol-containing backbone. DNA micro-array experiments established that the *tarIJKL* gene cluster is required for the biosynthesis of this alternative WTA backbone, and suggest ribose and arabinose are precursors thereof. Increased *tarIJKL* expression was not observed in any of our previously performed DNA microarray experiments, nor in qRT-PCR analyses of *L. plantarum* grown on various carbon sources, leaving the natural conditions leading to WTA backbone alditol switching, if any, to be identified. Human embryonic kidney NF-κB reporter cells expressing Toll like receptor (TLR)-2/6 were exposed to purified WTAs and/or the TA mutants, indicating that WTA is not directly involved in TLR-2/6 signaling, but attenuates this signaling in a backbone independent manner, likely by affecting the release and exposure of immunomodulatory compounds such as LTA. Moreover, human dendritic cells did not secrete any cytokines when purified WTAs were applied, whereas they secreted drastically decreased levels of the pro-inflammatory cytokines IL-12p70 and TNF-α after stimulation with the WTA mutants as compared to the wild-type.

**Conclusions:**

The study presented here correlates structural differences in WTA to their functional characteristics, thereby providing important information aiding to improve our understanding of molecular host-microbe interactions and probiotic functionality.

## Background

In Gram-positive bacteria the cytoplasmic membrane is covered by a thick cell wall consisting of multiple layers of peptidoglycan, an essential polymer composed of alternating residues of β-1-4-linked *N*-acetyl muramic acid (Mur*N*ac) and *N*-acetyl-glucosamine (Glc*N*ac) [1]. Several other molecules are present in the cell envelope, including teichoic acids (TAs), polysaccharides, and extracellular proteins [2-4]. Most Gram-positive bacteria produce two distinct types of TA that make up a substantial percentage of total cell envelop dry-weight; wall teichoic acid (WTA) is covalently anchored to the Mur*N*AC residue of peptidoglycan via a phosphodiester bond, whereas lipoteichoic acid (LTA) is anchored in the cytoplasmic membrane through a glycolipid [5,6]. LTAs typically consist of repeating units of glycerol-phosphate [7]. By contrast, WTAs containing a variety of different alditols have been reported [5], although glycerol and ribitol are most common [8].

**Figure 1:**
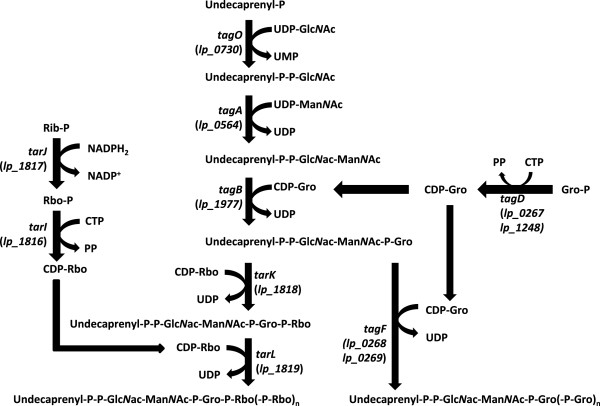
**Functions of the proteins encoded by the *tag *and *tar *genes in the biosynthesis of poly(Gro-P)- and poly(Rbo-P)-containing WTA backbones, respectively [adapted from **[9]**.** Numbers between brackets indicate the gene-identifiers of *tag* and *tar* homologues in the *L. plantarum* WCFS1 genome and Rib represents ribulose. Regardless of the backbone type, the biosynthesis of WTA is initiated with the consecutive coupling of UDP-activated *N*-acetylglucosamine (Glc*N*ac) and *N*-acetylmannosamine (Man*N*ac) to undecaprenyl phosphate on the cytoplasmic side of the cell membrane by TagO and TagA, respectively [5]. Gro-P is CDP-activated by TagD and coupled to this disaccharide linkage unit by the primase TagB. In strains producing poly(Gro-P) WTA the final biosynthetic step involves the coupling of a large but variable amount of CDP-activated Gro-P monomers by the oligomerase TagF. Although TagF homologues (sometimes designated TarF) are present in several strains producing poly(Rbo-P) WTA, these enzymes are generally smaller than their TagF counterparts and their activity is limited to the incorporation of 1 or 2 additional CDP-activated Gro-P monomers [9,62]. In the final biosynthetic steps CDP-activated Rbo-P, produced by TarJ and TarI, is added to the Gro-P mono-, di- or tri-mer by the primase (TarK) and the oligomerase activity (TarL), resulting in the addition of multiple repeating units of Rbo-P [5,9,13,63]. Following its synthesis, the complete WTA polymer is proposed to be transported across the cytoplasmic membrane by the *tagGH*-encoded ABC transporter, and is subsequently coupled to the 6-hydroxyl of Mur*N*Ac in peptidoglycan by an unknown transferase [5,13]. These basic WTA polymers can be substituted with D-alanyl esters through the activity of the enzymes encoded in the *dlt* operon [31,64,65] and/or glycosyl moieties by homologues of TagE or TarM of *B. subtilis* and *S. aureus*, respectively [39,40].

The *tag* and *tar* gene cluster products are responsible for WTA biosynthesis of the poly(glycerol-3-phosphate) [poly(Gro-P)] and poly(ribitol-5-phosphate) [poly(Rbo-P)] types, respectively. These genes have been extensively studied in *Bacillus subtilis*[9] and *Staphylococcus aureus*[5,10], allowing the identification of most gene functions involved in WTA backbone polymer biosynthesis (Figure 1) and transport, as well as D-Ala and glycosyl substitution of the basic polymer. Initially, construction of *tag* and *tar* gene deletion mutants appeared impossible and WTA was thought to be essential [11,12]. More recently, WTA deficient mutants were successfully obtained, e.g. in *S. aureus*[13,14], *Lactobacillus plantarum*[15], and *B. subtilis*[16], by deletion of the first gene in the biosynthetic pathway (*tagO*). This suggests that earlier attempts to disrupt genes which function at a later stage in the pathway result in the accumulation of toxic intermediates or the depletion of components like undecaprenyl phosphate that typically function as a scaffold in cell-wall component biosynthesis [1,17]. Phenotypic analysis of the *L. plantarum* and *B. subtilis tagO* mutants indicated an important role for WTA in normal cell elongation and cell-shape maintenance [15,18]. 

The lactobacilli studied to date have all been shown to produce poly(Gro-P) WTA molecules [17], although some species have been identified that do not produce any WTA, i.e. *L. rhamnosus, L. casei*, *L. fermentum* and *L. reuteri*[2,3]. Moreover, *L. plantarum* strains appear to produce either poly(Gro-P) or poly(Rbo-P) WTA [3,8]. Recently, *L. plantarum* strains, including the most studied strain WCFS1, that encode the *tagD1-tagF1-tagF2* genes (*tag*-locus) were shown to consistently produce poly(Gro-P) WTA, while strains that lack these genes produce poly(Rbo-P) WTA [8]. The production of poly(Rbo-P) WTA in *L. plantarum* was proposed to depend on the *tar* locus (*tarIJKL*) that is universally conserved among *L. plantarum* strains [8] and was originally annotated as *lp_1816-lp_1817-tagB1-tagB2* in the genome sequence of strain WCFS1 [19]. Despite the fact that poly(Gro-P) WTA producing *L. plantarum* strains consistently encode both the *tag* and *tar* loci, and therefore possess the genetic capability to synthesize WTA with either backbone alditol, there have been no reports of strains that produce the Rbo-P or both WTA subtypes, indicating that the presence of the *tag*-locus exclusively dictates WTA production [8].

Following consumption, *L. plantarum* strains typically survive for several days in the human gastrointestinal tract [20] and adhere to human mucosa [21]. Specific strains of *L. plantarum*, have demonstrated health-promoting effects on the consumer [22]. For example, consumption of *L. plantarum* strain WCFS1 was shown to trigger mucosal gene expression patterns and cellular pathways that correlate with the establishment of immune tolerance in healthy adults [23]. The same study suggests that the molecular host responses towards *L. plantarum* WCFS1 in human intestinal mucosa depends on the cell-envelope composition of the culture administered [23]. Moreover, there is compelling evidence that specific *Lactobacillus* cell-wall components play a pivotal role in the interaction of probiotic lactobacilli with the immune system, e.g. specific tripeptides derived from peptidoglycan modulate immune responses via human NOD-like receptor 1 and 2 (NOD1 and NOD2, [3,24-26]). Furthermore, TLR-2 in association with TLR-1 or TLR-6 plays an important role in the innate immune response by recognizing microbial lipoproteins and lipopeptides [26,27]. To this end, LTA of several *Lactobacillus* species [28-30], including *L. plantarum* NCIMB8826 (of which strain WCFS1 is a single colony isolate) [31], was demonstrated to interact with Toll like receptor 2 (TLR-2, [3,4,26]). Moreover, purified LTA from *L. plantarum* WCFS1 was shown to elicit the production of the cytokine tumor necrosis factor alpha (TNF-α) in murine bone-marrow cells in a TLR-2 dependent manner, which is largely dependent on D-Ala substitution of the LTA backbone [31].

In contrast to LTA, the role of WTA in TLR-2-mediated immune signaling is debated [3,32], especially as WTA is not acylated [5] which is considered an essential characteristic for LTA signaling [26]. Moreover, contamination of purified WTA fractions with other immunomodulating bacterial components may have blurred the assignment of the reported effects to WTA-mediated signaling. For example the WTA of *L. casei* Shirota has been proposed to act synergistically with LTA to induce IL-10 production through a TLR-2-dependent pathway [33], while genome comparisons of genetic loci involved in WTA biosynthesis suggest that *L. casei* strains cannot produce WTA [2]. Here, we aimed to clarify the role of WTA by addressing the following questions; is *L. plantarum* WCFS1 capable of switching its WTA backbone alditol? Can the immune system recognize *L. plantarum* poly(Gro-P) and/or poly(Rbo-P) WTA in addition to LTA?

The fact that some *L. plantarum* strains possess the genetic capacity to produce both WTA-backbone types, offers the possibility to study the physiological consequences and host-signaling induced by these WTA variants in an isogenic background. Therefore, we biochemically analyzed the modified WTA synthesized by *L. plantarum* WCFS1 mutants demonstrating that (i) a *tagO* deletion mutant is unable to produce WTA, (ii) a *tagF1-2* deletion mutant produces WTA with a poly(Rbo-P) backbone instead of poly(Gro-P), and (iii) a *dltX-D* deletion mutant lacks D-Ala substitution of both LTA and WTA. Subsequently, DNA microarrays revealed the consequences of these modifications on bacterial physiology and the WTA biosynthetic pathways. To gain insight into the potential role of WTA and its modifications on host-immune interactions, the differential effect of the mutants and purified WTA derived from these mutants on cytokine production by human monocyte derived dendritic cells, as well as their innate signaling capacity via TLR-2/6 in a NF-κB reporter cell line, was investigated.

## Results

### *in silico* analysis of the WTA biosynthetic pathways in *L. plantarum*

**Figure 2:**
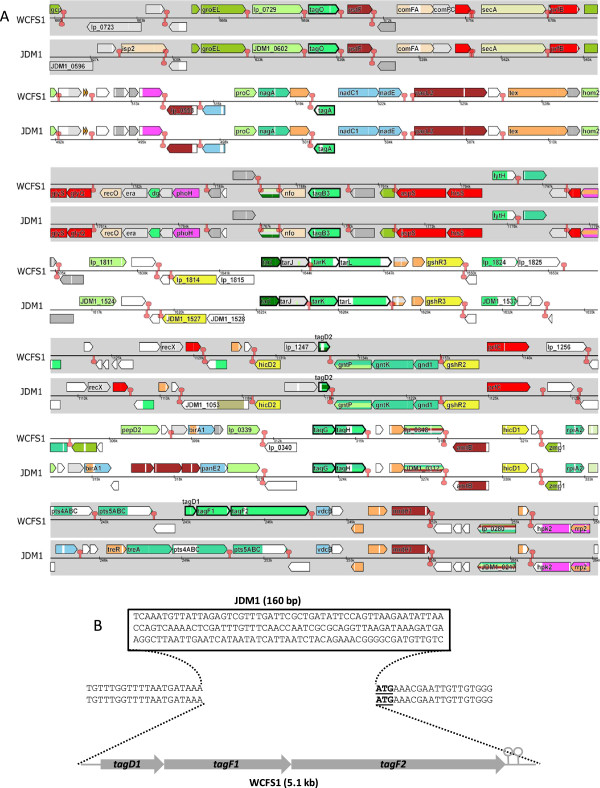
**Chromosomal organization of the 7 distinct genetic loci (represented by the iterating grey- and unshaded boxes) harboring the *tag *and *tar *genes in the *L. plantarum *WCFS1 and JDM-1 genomes (panel A).** The *tag* and *tar* genes are highlighted in bold and named according to the current annotation in the WCFS1 genome. Colors represent different functional classes as defined by the microbial genome viewer tool [66]. Panel **B** displays detailed sequence information around the *tagD1-tagF1-tagF2* locus (last grey-shaded box in Panel A) with the start codon of the downstream-encoded *lp_0271* (*vdcB*) underscored.

The NCBI database ( http://www.ncbi.nlm.nih.gov/) contains the complete and annotated genome sequences of the *L. plantarum* strains WCFS1 [19,34] and JDM-1 [35]. By using blastn [36], it was established that both genomes contain homologues of *tagO*, *tagA*, *tagD*, *tagB, tarIJKL* (*tar*-locus), and *tagGH* (Figures 1 and 2A). In contrast to *B. subtilis*[9] and *S. aureus*[10], the *L. plantarum* genes required for biosynthesis and transport of WTA backbones are scattered over 6 chromosomal loci, with a conserved chromosomal organization in *L. plantarum* WCFS1 and JDM-1. Only the WCFS1 genome contains the additional *tag*-locus (*tagD1F1F2*) (Figure 2A and B) that was previously predicted to dictate the production of poly(Gro-P) WTA in this strain, while the absence of this locus in strain JDM1 would predict this strain to produce the poly(Rbo-P) WTA variant [8]. At the chromosomal location that harbors the WCFS1 *tag*-locus, the JDM1 chromosome contains a stretch of 160 bp that is not present in the WCFS1 genome (Figure 2B). 

### Genetic engineering results in the biosynthesis of alternative WTA variants

Gene deletion mutants were exploited to assess the capability of *L. plantarum* WCFS1 to produce alternative WTA variants. To study the consequences of WTA removal, we used a recently described mutant in which the single copy of *tagO* was deleted [15]. Notably, the LTA backbone is synthesized via a completely independent pathway and, therefore, its biosynthesis is anticipated to be unaltered in *tagO* mutants [5]. In addition, the Gro-P polymerases (encoded by *tagF*) were targeted by mutagenesis, thereby anticipating to block the production of a poly(Gro-P)-containing WTA backbone (Figure 1), while maintaining the capability to synthesize a Gro-P-containing linkage unit (TagB activity) that is likely to be also involved in biosynthesis of a poly(Rbo-P) WTA [5,9,37]. Although two genes are annotated as *tagF*, the size difference between the encoded TagF1 (lp_0268, 402 residues) and TagF2 (lp_0269, 941 residues) suggests that TagF1 might in fact encode an additional Gro-P primase (TagB) [9]. Nevertheless, as the function of TagF1 is currently unestablished, a *tagF1-F2* mutant that lacks both genes was constructed. Lastly, a stable mutant was constructed in which the complete *dltX-D* operon was deleted, aiming to produce WTA (and LTA [31]) that lacks D-Ala substitution. Notably, an *L. plantarum* mutant unable to substitute LTA with D-Ala residues has previously been described. However, this mutant harbored a *dltB* gene disruption obtained by single cross over plasmid integration within the *dltB* coding region, and as a consequence is genetically unstable [31]. Importantly, our *dltX-D* mutant displayed identical alterations in its basic physiological characteristics as the previously constructed *dltB* mutant [31,38], including a lower maximum growth rate and final biomass, as well as cell lysis after prolonged stationary phase incubation (data not shown). Furthermore, the *tagO* mutant was already reported to display the tendency to remain associated in chains, and its cells appeared swollen, bent, and/or shorter than the wild-type [15]. By contrast, the growth and morphological characteristics of the newly constructed *tagF1-2* mutant closely resembled the wild-type strain (data not shown).

**Figure 3:**
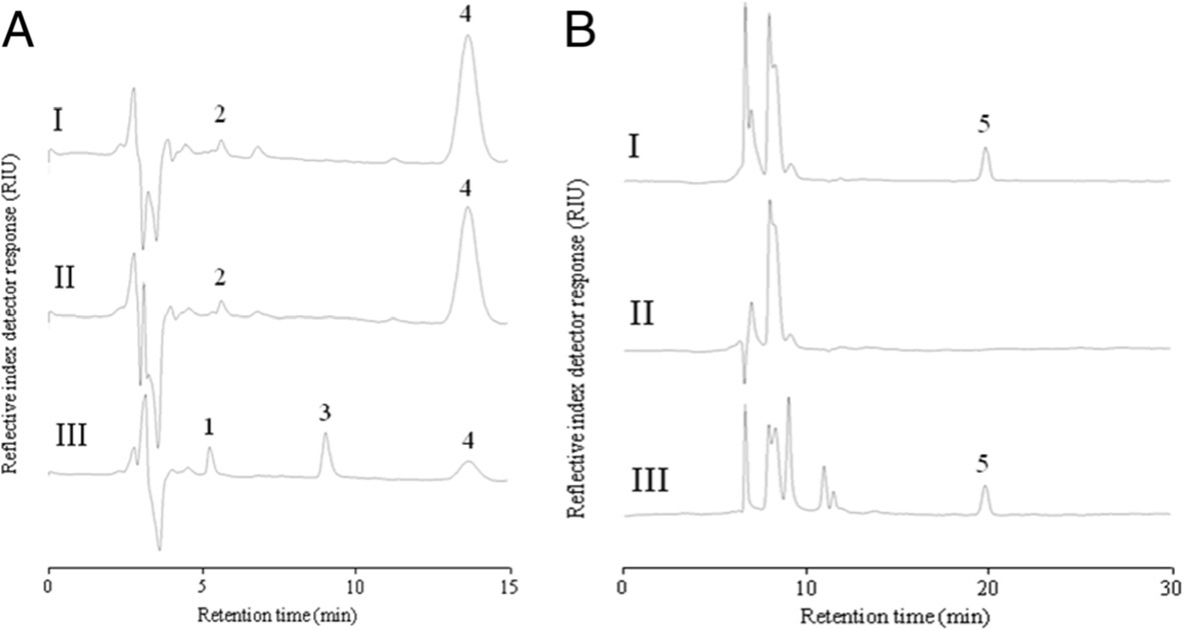
**Sugars, alditols, and D-Ala composition of WTA isolated from *L. plantarum *WCFS1 (I), and the *dltX-D *(II), and *tagF1-F2 *(III) mutants.** Panel **A** represents chromatograms on a Shodex NH2P-50 4E column, whereas panel **B** displays chromatograms on a Shodex SUGAR SC1011 column. Using pure compounds, peaks 1, 2, 3, 4 and 5 were identified to represent anhydroribitol, glycerol, ribitol, glucose, and alanine, respectively. The experiment is representative of 2 HPLC analyses.

To assess alditol composition, as well as glucosyl and D-Ala content, WTA was purified from the wild-type *L. plantarum* WCFS1 strain, and its *tagO*, *tagF12*, and *dltX-D* gene deletion derivatives (Figure 3 and Table 1). No WTA could be isolated from the *tagO* mutant, confirming earlier observations [15] that disruption of the first gene in the WTA biosynthetic pathway completely blocks WTA production in *L. plantarum*. From the other strains WTA isolation was successful and their phosphoric content was highly comparable, ranging from 2.0-2.3 μmol mg^-1^ (Table 1). The chemical composition analysis of WTA purified from the wild-type and the *dltX-D* mutant established the presence of a poly(Gro-P) backbone (Figure 3), as well as the presence of glucosyl moieties (Table 1, 2.3 and 2.4 μmol mg^-1^, respectively). In contrast to the wild-type (0.79 μmol mg^-1^), no D-Ala could be detected in the purified WTA obtained from the *dltX-D* mutant, establishing the role of *dltXD* in D-alanylation of *L. plantarum* WTA next to its previously described role in D-Ala substitution of LTA [31]. The chemical analysis of WTA purified from the *tagF12* mutant completely lacked the glycerol-specific signal, whereas (anhydro)ribitol could clearly be detected (Figure 3). These data demonstrate that the *tagF1-2* mutant of *L. plantarum* WCFS1 is capable of producing WTA containing a poly(Rbo-P) backbone as an alternative for the poly(Gro-P) backbone produced by the parental strain. Notably, the glucosyl content (1.1 μmol mg^-1^) and D-alanylation (0.57 μmol mg^-1^), of this alternative WTA produced by the *tagF12* mutant appeared to be reduced in comparison with the native poly(Gro-P) WTA produced by the wild-type. In addition, the relative abundance of poly(Rbo-P) WTA in the *tagF12* mutant (32% of total cell wall weight) was reduced compared to that of the poly(Gro-P) WTA in the wild-type (51%) and *dltX-D* mutant (51%) strains. Overall, the genetic engineering approach employed allowed the production of WTA variants in an isogenic *L. plantarum* background, and corroborates the inferences drawn from the *in silico* analysis. 

**Table 1:** **Quantitative WTA analysis of *L. plantarum *WCFS1, and the *tagF1 *- *2 *and *dltX-D *mutants**

	**Phosphorus content (μmol mg**^**-1**^**):**						
**Strain:**	**WTA:**	**Cell wall:**	**% WTA (w/w):**	**D-Ala (μmol mg**^**-1**^**):**	**D-glucose (μmol mg**^**-1**^**):**	**D-Ala / P:**	**D-glucose / P:**
wild-type	2.0	1.0	51	0.79	2.3	0.39	1.1
Δ*dltX-D* (NZ3539Cm)	2.3	1.2	51	N.D.	2.4	0.0	1.0
Δ*tagF1*-*2* (NZ3547Cm)	2.2	0.72	32	0.57	1.1	0.25	0.51

N.D. = not detectable (<0.01 μmol mg^-1^). The data is representative for 2 independent HPLC analyses.

To assess whether the production of alternative WTA molecules by the mutants impacts on either WTA or LTA polymer chain-length, purified WTA and LTA were utilized for high performance size exclusion chromatography (HPSEC) and proton NMR analyses, respectively (see the Additional file 1). These analyses reconfirmed that no WTA was produced by the *tagO* mutant, whereas the WTA produced by the *dltX-D* mutant was of virtually identical backbone polymer length as compared to the WTA produced by the wild-type (Additional file 1: Table S1 in the supplementary information file). The WTA produced by the *tagF1-2* mutant appeared to contain approximately 25% less backbone units (Rbo-P) as compared to the wild-type (Gro-P units). Similarly, LTA derived from the *tagO* and *dltX-D* mutants harbor a similar amount of Gro-P backbone units as compared to the wild-type, whereas the Gro-P LTA isolated from the *tagF1-2* mutant appeared approximately 25% lower (Additional file 1: Table S1 in the supplementary information file). Taken together, these data suggest that the described effects of WTA-pathway engineering have relatively small effects on backbone chain length of WTA and LTA.

### Transcriptome analyses of mutants producing altered WTA

To assess the impact of the production of the alternative WTA molecules on overall cell physiology, and specifically the WTA biosynthetic routes, whole-genome transcriptome profiles were generated for the *dltX-D* and *tagF1-2* mutants and compared to the wild-type (Table 2 for genes involved in WTA biosynthesis and Additional file 2: Table S2 for all significantly regulated genes). As anticipated, no significant expression of the *tagF1-2* and *dltX-D* genes could be detected in the corresponding mutants, verifying the expected mutation (Table 2). Remarkably, 230 and 402 genes appeared to display at least 2-fold altered transcription levels in the *dltX-D* and *tagF1-2* mutants, including 30 and 47 genes that are annotated in the main functional class “cell envelope”, respectively (Additional file 2: Table S2). These data strongly suggest compensatory behavior of *L. plantarum* upon modification of the cell wall component WTA (either lack of D-alanylation or alditol backbone switching), emphasizing the pivotal role WTA plays in the overall cell physiology of *L. plantarum*[15]. 

**Table 2:** **Relative transcription levels of genes involved in WTA biosynthesis and D-Ala and glycosyl substitution in the *dltX-D *(NZ3539Cm) and *tagF1-2 *(NZ3547Cm) mutants as compared to the wild-type strain**

**lp number:**	**Gene name:**	**NZ3539Cm/WCFS1:**	**fdr:**	**NZ3547Cm/WCFS1:**	**fdr:**
*lp_0267*	*tagD1*	1.139	0.495	**0.302**	**0.000**
*lp_0268*	*tagF1*	1.274	0.470	**0.003**	**0.000**
*lp_0269*	*tagF2*	1.554	0.421	**0.000**	**0.000**
*lp_0343*	*tagG*	**2.667**	**0.000**	**2.394**	**0.001**
*lp_0344*	*tagH*	**3.386**	**0.000**	**2.380**	**0.001**
*lp_0564*	*tagA*	1.814	0.013	1.501	0.120
*lp_0730*	*tagO*	1.071	0.921	**3.243**	**0.000**
*lp_1248*	*tagD2*	1.163	0.476	1.228	0.324
*lp_1816*	*tarI*	**6.638**	**0.006**	**317.9**	**0.000**
*lp_1817*	*tarJ*	**7.145**	**0.004**	**390.0**	**0.000**
*lp_1818*	*tarK*	1.253	0.452	**15.42**	**0.000**
*lp_1819*	*tarL*	2.291	0.067	**48.23**	**0.000**
*lp_1977*	*tagB3*	1.107	0.964	0.885	0.631
*lp_2016*	*dltD*	**0.001**	**0.000**	0.792	0.245
*lp_2017*	*dltC1*	**0.002**	**0.000**	**0.545**	**0.031**
*lp_2018*	*dltB*	**0.001**	**0.000**	0.855	0.504
*lp_2019*	*dltA*	**0.068**	**0.000**	**0.286**	**0.006**
*lp_2020*	*dltX*	**0.207**	**0.000**	0.905	0.706
*lp_1406*	*dltC2*	1.157	0.617	0.540	0.068

Bold numbers represent significantly changed transcripts levels (fdr < 0.05).

**Figure 4:**
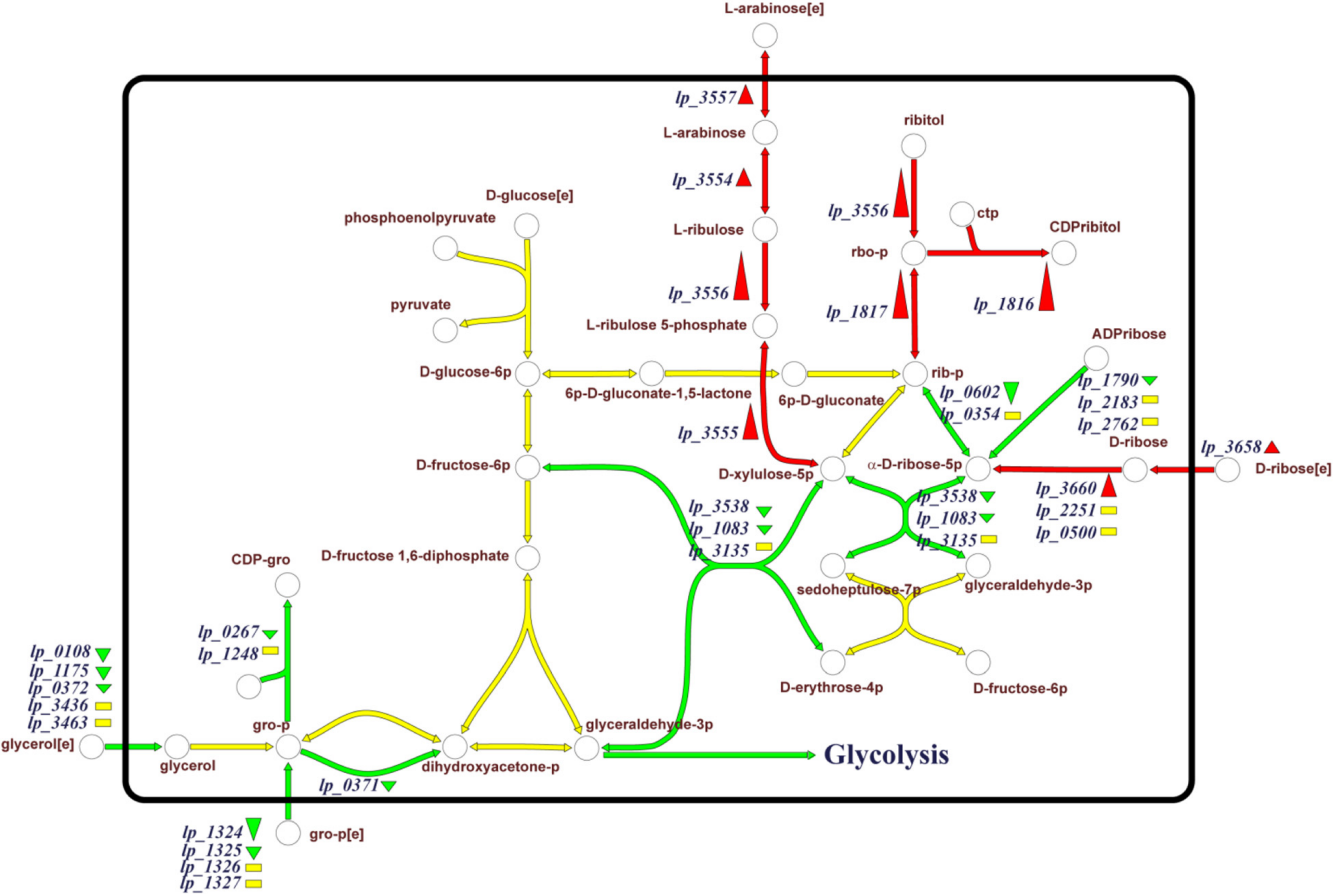
**Simpheny visualization map (www. genomatica.com) using the *L. plantarum *specific model **[67]**of the biosynthetic pathways involved in WTA backbone precursor biosynthesis.** Red and green arrow(head) represents induced and repressed expression levels as assessed by DNA microarrays, whereas yellow arrows and boxes indicate unchanged expression levels in the *L. plantarum tagF1-2* mutant as compared to the wild-type, respectively. The size of the arrowheads reflects the quantitative induction/repression levels, the black line represents the cytoplasmic membrane, and [e] indicates extracellular localization.

Interestingly, the *dltX-D* mutation led to a 3-fold induced expression of the *tagGH*-encoded WTA transporter (Table 2). In addition, this mutant strain also expressed the *tar*-locus (*tarIJKL*) at a 7-fold higher level as compared to the wild-type, suggesting that the *dltX-D* mutant may produce minute quantities of the poly(Rbo-P) WTA that remained undetected in the biochemical analyses, next to the dominant poly(Gro-P) WTA. When compared to the wild-type, the *tarIJKL* operon appeared among the strongest induced transcripts (up to 390-fold) identified in the transcriptome of the *tagF1-2* mutant (Table 2). This finding in combination with the *in silico* and biochemical analyses described above establishes a definite role for *tarIJKL* in the production of the alternative poly(Rbo-P) WTA backbone in *L. plantarum* WCFS1. Notably, an ABC-transporter encoded by *lp_0298*-*lp_0299* represents another genetic locus that displays strongly elevated expression levels in the *tagF1-2* mutant as compared to the wild-type (Additional file 2: Table S2, 291- and 1049-fold, respectively). Although the substrate for this transporter is unknown, its elevated expression might indicate that it is involved in import of Rbo-P precursors or export of the poly(Rbo-P) WTA. In addition, several genes involved in production of the linkage unit (*tagO*, *tagA*, and *tagD2*) and the TA-transporter, predicted to be encoded by *tagGH*, appeared induced in the poly(Rbo-P) WTA producing *tagF1-2* mutant (Table 2). Despite the fact that these *tag* and *tar* genes displayed elevated transcription levels in the *tagF1*-*2* mutant, the amount of WTA this strain produced is lower than that of the wild-type (see above), suggesting a lower overall efficacy of poly(Rbo-P) WTA biosynthesis and biogenesis as compared to its poly(Gro-P) counterpart. In addition, the *dltX-D* operon expression level appeared lower in the *tagF1-2* mutant, which is in agreement with the reduced level of D-Ala substitution of the poly(Rbo-P) WTA relative to the poly(Gro-P) WTA produced by the wild-type. The fact that poly(Rbo-P) WTA biosynthesis utilizes alternative precursors was also clearly reflected in the transcriptome profiles. For example, the expression of the Gro-P-ABC transporter (*lp_1324-1327*), as well as *tagD1* (*lp_0267*), involved in UDP activation of Gro-P, was decreased in the *tagF1-2* mutant (Figure 4). Moreover, virtually all genes responsible for the import and conversion of both ribose and arabinose to CDP-Rbo displayed increased expression levels in the *tagF1-2* mutant, whereas several of the surrounding pathways bridging to synthesis of other sugar-intermediates and glycolysis were down-regulated (Figure 4).

The *tagE* genes encoded in the *L. plantarum* genome might be involved in glucosyl substitution of the poly(Rbo-P) WTA, based on their homology to TagE and TarM of *B. subtilis* and *S. aureus*, respectively [39,40]. This notion is supported by the approximately 1.8-fold increased expression levels of *tagE1*, *tagE3*, and *tagE6* homologues, in the *tagF1-2* mutant. In addition, several other genes annotated as glycosyltransferases were either up- (*lp_2845* and *lp_3050*; 17- and 5-fold, respectively) or down-regulated (*lp_0304* and *lp_1763*; both 4-fold). This indicates that an additional or complementary role of these genes, but also glysosyl-transferases that displayed unaltered expression levels, in glycosyl substitution of either of the two WTA variants can certainly not be excluded.

### WTA backbone switching does not occur under laboratory conditions

The experiments above unambiguously establish mutation of the *tagF1-2* locus in *L. plantarum* WCFS1 can induce the biosynthesis of WTA containing the alternative poly(Rbo-P) backbone alditol, while the hyperinduction of the *tarIJKL* locus is a genetic marker for this event. Importantly, sequence analysis of the promoter upstream of the *tarIJKL* operon in the *tagF1-2* mutant revealed the induced transcription of *tarIJKL* is not caused by promoter mutation, implying that the regulatory mechanism for WTA backbone alditol switching is intact in *L. plantarum* WCFS1. Therefore, it might be possible to induce WTA backbone alditol switching by modification of environmental conditions during growth. Hence, we attempted to identify fermentation conditions that lead to natural WTA alditol backbone switching. Firstly, we analyzed 29 transcriptome profiles generated previously in our laboratory in which varying temperature and pH were used, as well as different concentrations of amino acids, oxygen, and NaCl [41]. However, expression of the *tarIJKL* locus appeared extremely low under all these conditions (Additional file 3: Figure S1), suggesting that *L. plantarum* WCFS1 consistently produces the poly(Gro-P)-containing WTA backbone in these fermentations previously performed in our laboratory. Secondly, the observation that the transcriptome profiles generated for the *tagF1-2* mutant (see above) revealed the induction of virtually all genes involved in the import and conversion of ribose and arabinose (Figure 4), triggered us to ferment *L. plantarum* WCFS1 using alternative sugars as carbon sources. Cells were harvested from logarithmic phase after growth on sucrose or ribose as sole carbon source, and RNA was isolated for qRT-PCR analyses, revealing low *tarIJKL* expression levels, highly comparable to *L. plantarum* grown on glucose which is established to produce a Gro-P-containing WTA by our biochemical analyses. Notably, bacterial growth on arabinose was insufficient to perform accurate qRT-PCR analysis of the *tar* locus. Therefore, the natural conditions resulting in WTA backbone alditol switching, if any, remain to be established. Taken together, these data indicate that the regulatory circuit required for the WTA alditol backbone switch is functional in *L. plantarum* WCFS1, but the natural conditions leading to alditol-switching remain to be identified. In the *tagF1-2* mutant background 35 regulators were differentially expressed compared to the wild type strain (Additional file 2: Table S2), suggesting that one or more of these regulators may play a role in the regulation of alditol-switching of the WTA biosynthesis pathway.

### The role of WTA in immune signaling and modulation

**Figure 5:**
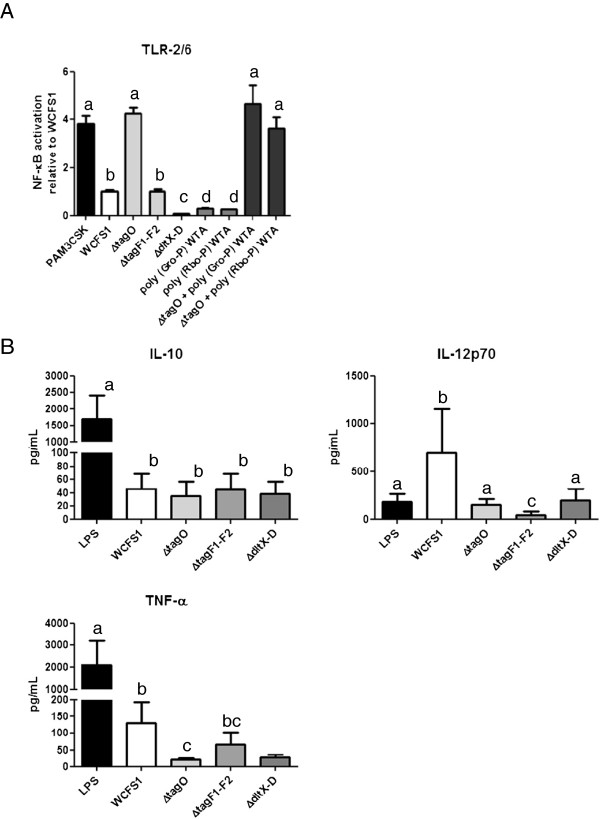
**Immunomodulating and signaling capacity of the *L. plantarum *TA mutants.** Panel **A** displays NF-κB pathway activation relative to *L. plantarum* WCFS1, as measured by a luminescence reporter in HEK cell lines expressing TLR-2/6 after exposure to PAM3CSK (positive control), purified WTA (5.0 μg/ml, see Additional file 4: Figure S2 and Additional file 5: Figure S3 for concentration ranges) and/or *L. plantarum* WCFS1, the *tagO*, *tagF1*-*2,* or *dltX-D* mutants. The ratios of HEK reporter to bacterial cell were 1:15. All strains were grown in triplicate and each culture was applied three times to the assay. Bars represent averages of these nine measurements with standard deviations. Letters a-d represent classes of statistically different responses as determined by ANOVA multiple comparison. Panel **B** displays cytokine levels secreted by monocyte derived iDCs stimulated with LPS (positive control), *L. plantarum* WCFS1, and the *tagO*, *tagF1*-*2* and *dltX-D* mutants. The ratios of dendritic to bacterial cells were 1:10. All strains were grown in duplicate and each culture was applied in duplicate to the assay. Bars represent averages of these four measurements with standard deviations. Individual letters (a-c) represent classes of statistically significant different responses as determined by restricted maximum likelihood analysis, whereas “bc” represents a class not significantly different from “b” nor “c”.

To identify the role of TLR-2/6 heterodimers in WTA immune signaling, the NF-κB pathway activating capacity of purified WTAs and the *L. plantarum* TA mutants were assessed in a HEK-reporter cell line that expresses TLR-2/6. Exposure of this HEK-reporter cell line to wild-type *L. plantarum* cells activated TLR2/6 induced NF-κB, whereas virtually no activation was measured with the *dltX-D* mutant (Figure 5A), which resembles earlier observations by Grangette *et al.*. using a *L. plantarum dltB* mutant and purified LTA [31], confirming the suitability of our signaling assay. These experiments indicate that TLR-2/6 signaling by intact *L. plantarum* WCFS1 cells depends on the presence of D-Ala substituted TAs on the cell surface. Furthermore, addition of a wide concentration range (0.2-125 μg/ml) of WTA purified from the wild-type [poly(Gro-P) backbone] and the *tagF1-2* mutant [poly(Rbo-P) backbone] in the assays did not result in any signal above the medium control (Figure 5A for an exemplary WTA concentration and Additional file 4: Figure S2 for a large range of WTA concentrations), demonstrating that WTA has no direct role in TLR-2/6 signaling. Furthermore, TLR-2/6-mediated NF-κB activation by the *tagF1-2* mutant was similar to the wild-type (Figure 5A), whereas the *tagO* mutant displayed significantly increased signaling, suggesting that WTA deficiency might lead to increased exposure of TLR-2/6 agonists such as LTA, independent of the WTA backbone alditol. Attempts to functionally complement the *tagO* mutant by the addition of purified poly(Gro-P) or poly(Rbo-P) WTA, followed by exposure to the HEK-reporter cell line, revealed enhanced signaling, highly similar to addition of the *tagO* mutant alone (Additional file 5: Figure S3, Figure 5), indicating that WTA is only capable of shielding TLR-2/6 signaling when it is localized appropriately in the cell wall. 

To evaluate the immunomodulating characteristics of WTA, the cytokine production profiles of immature human monocyte derived dendritic cells (iDCs) were determined after stimulation with purified WTA and the mutant strains. Application of purified poly(Gro-P) or poly(Rbo-P) WTA to this assay resulted in undetectable interleukin (IL)-10, IL-12p70 and TNF-α secretion levels, comparable to iDCs to which medium was applied as a control (<0.1 pg ml^-1^, data not shown), suggesting that WTA is not directly involved in immunomodulation. The IL-10 levels secreted by DCs stimulated with all TA mutants were comparable to the levels obtained with the wild-type (Figure 5B). By contrast, levels of IL-12p70 were significantly lower for the *dltX-D* mutant as compared to the wild-type, suggesting that D-Ala substitution of TAs strongly contributes to the capacity of *L. plantarum* to elicit pro-inflammatory immune responses. The *tagO* mutant also elicited a decreased production of IL-12p70 as compared to the wild-type, indicating that, in addition to LTA [31], the presence of WTA impacts on IL-12p70 production levels. Moreover, the IL-12 production levels obtained upon exposure of immature DCs (iDCs) to the *tagF1-2* mutant were even lower than those of the *dltX-D* and *tagO* mutants, indicating that the biosynthesis of the alternative poly(Rbo-P)-containing WTA backbone has differential consequences for the immune response elicited by DCs, resulting in altered pro-inflammatory cytokine production. The observed reduction of IL-12 production levels in these DC assays was reflected (although significant only for *tagO* mutant) in the levels of TNF-α production in these assays.

## Discussion

Several *L. plantarum* strains encode the genetic determinants for the production of WTA variants containing either poly(Gro-P) or poly(Rbo-P) backbones [8], whereas other lactobacilli appear to exclusively produce the poly(Gro-P) variant or produce no WTA at all [2,3]. LTA polymers consistently contain a glycerol backbone [29] that is synthesized via an independent pathway involving LtaS (LTA synthase)-dependent polymerization of phosphatidylglycerol, rather than the nucleotide-activated sugars utilized as precursors for WTA biosynthesis [5]. Moreover, LTA phosphatidylglycerol polymerization occurs directly onto a glycolipid that serves as the membrane anchor, rather than the undecaprenyl phosphate anchor employed in WTA biosynthesis [5,18]. Indeed, we were able to show here that modification of WTA had no impact on the type of LTA backbone produced (GroP), and only slightly altered the chain length in the case of the *tagF1-2* mutant. The independence of these biosynthetic pathways allowed investigation of WTA-specific alditol switching in an isogenic background in *L. plantarum* WCFS1, as well as the assessment of the overall effects thereof on bacterial physiology and the WTA biosynthetic routes using a transcriptomics approach. In view of the substantial transcriptome responses elicited by the mutations introduced, it may be that TA engineering has some general effects for cell wall architecture and turn-over that go beyond the specific components that were targeted by mutagenesis here. This may elicit some effects in terms of host cell recognition and/or response, but the data presented clearly establish that immunomodulatory effects observed in this study are not directly influenced by the WTAs.

The work presented here functionally demonstrates a role for the *tar*-locus (*tarIJKL*) in the production of poly(Rbo-P) WTA in *L. plantarum.* Moreover, the DNA microarray experiments performed with *L. plantarum* WCFS1 and its derivatives producing alternative WTA variants indicate that ribose and/or arabinose may serve as the preferred carbohydrate sources for the production of the precursors for this alternative WTA backbone. However, we have not been able to identify fermentation conditions that lead to the natural switching of the WTA backbone. Reports on other Gram-positive bacteria such as *B. subtilis* and *S. aureus* describe the production of strain-specific WTA backbone alditols [5], as well as an *S. aureus* strain producing both poly(Gro-P) and poly(Rbo-P) WTA types simultaneously [42]. However, to the best of our knowledge, this is the first report presenting the genetic and functional capability of WTA alditol-backbone switching. Moreover, it also is the first study that analyzes the biosynthetic WTA pathway(s) in a probiotic species and evaluates the consequences for modulation of host immune cells and innate, TLR-2/6-mediated signaling.

A comparative *in silico* analysis of the poly(Gro-P) and poly(Rbo-P) associated WTA genes in the *B. subtilis* strains W23 and 168, respectively, suggests that at least some of the genes encoding poly(Gro-P) synthesis in strain 168 were acquired by horizontal gene transfer, possibly displacing all, or part of, the resident W23-like poly(Rbo-P) WTA biosynthesis genes [9]. Our *in silico* analysis suggested that a similar horizontal gene transfer event may have occurred in *L. plantarum*, whereby the genes involved in poly(Gro-P) WTA biosynthesis were acquired in an ancestral, poly(Rbo-P) WTA producing strain, while leaving the capacity to produce this native WTA variant intact. However, phylogenetic analysis of 18 *L. plantarum* strains revealed that the *tarIJKL* locus cluster in two groups correlating completely with the WTA backbone-type produced [8]. As it seems unlikely that the genes involved in poly(Rbo-P) WTA biosynthesis have co-evolved in strains producing poly(Gro-P) WTA, the exact order of evolutionary events remains unknown. Nevertheless, these events have led to a subset of *L. plantarum* strains that have the genetic potential to alternate their WTA biochemistry, which supports earlier suggestions that this canonical compound might play an important role in the lifestyle of this bacterium and may have a profound impact on its molecular communication with mucosal cells in the intestinal tract, i.e. its probiotic efficacy [2]. The observation that more poly(Gro-P) WTA is produced than poly(Rbo-P) WTA might present another possible explanation for the alditol switching, i.e. to produce higher quantities of this cell wall polymer under certain (yet unknown) stress conditions, possibly including adaptation to the host intestinal environment. To this end, it might also be interesting to investigate WTA alditol backbone switching by introducing the *tag* locus into strains producing Rbo-P type WTA.

The ability of peptidoglycan and other non-acylated cell wall components to induce TLR-2 signaling is controversial [43,44], and some studies have highlighted contamination with lipoproteins as a confounding factor [45]. Notably, these studies typically investigated immunomodulation by analyzing purified components of the cell envelope, ignoring shielding effects [46] as well as the interplay between different components, which is highly likely to blur our view of the full immunomodulatory capacity of intact bacterial cells possessing their native extracellular architecture and composition. Therefore, we assessed the differential immune responses triggered by exposure to *L. plantarum* TA mutants and/or purified WTA, thereby conclusively showing that purified *L. plantarum* WTA of either backbone alditol type is unable to directly signal via TLR-2/6. Nevertheless, the WTA negative derivative displayed enhanced TLR-2/6 signaling, suggesting that WTA shields or prevents release of other immunomodulating compounds, such as LTA, a known agonist of TLR2/6, and thereby indirectly contributes to attenuation of TLR-2/6 signaling. In addition, our observation that deletion of the genes required for D-alanylation of TA specifically reduces TLR-2/6 signaling, strongly suggests the TLR-2/6 heterodimer is involved in *L. plantarum* LTA signaling, in line with earlier experiments performed with Gram-positive pathogens [47,48]. Hence, our data fine-tune earlier murine-based observations revealing that *L. plantarum* LTA signaling is TLR-2-dependent [31], to the exact human TLR heterodimer involved. Notably, the *dltX-D* mutant reported here produced LTA of a backbone chain length highly comparable to *L. plantarum* WCFS1, whereas the previously constructed *dltB* mutant mainly produced LTA that was approximately 3 times longer than that produced by the wild-type [38]. Although the reason for this apparent discrepancy is currently unknown, it may be explained by the different nature of the genetic mutation introduced in these strains. While the mutant presented in this study lacks the entire gene cluster encoding the Dlt machinery, the mutant described by Palumbo *et al.*. carries a mutation only in the *dltB* gene. As a consequence, the remaining Dlt functions are likely still expressed in the latter mutant, which may influence the chain length determination of LTA, possibly by binding of Dlt components to the emerging LTA polymer. On the other hand, our results using the *dltX-D* mutant do corroborate a recent report that deletion of a phosphoglycerol transferase gene (*ltaS*), that plays a key role in LTA biosynthesis, in *Lactobacillus acidophilus* NCFM down-regulated IL-12 and TNF-α production in DCs in a TLR-2-dependent manner [30]. Conversely, the fact that the enhanced TLR-2/6 signaling of the *tagO* mutant observed in the HEK-reporter cell line that only expresses TLR-2/6 did not translate to elevated IL-12 and TNF-α levels in DCs equipped with the full arsenal of receptors, but in fact resulted in decreased levels of these cytokines, strengthens the concept that communication between probiotic lactobacilli and intestinal host cells is multi-factorial and involves an integrative repertoire of receptors on the host side that recognize multiple effector molecules on the bacterial side [49]. It remains to be established whether WTA is directly involved in signaling via other host receptors or, alternatively, shielding of immunomodulating compounds that signal via other receptors.

## Conclusions

The data presented here has several consequences for research addressing molecular host-probiotic communication. The observation that the pro-inflammatory capacity (IL-12 levels) of *L. plantarum* WCFS1 depends on the amount and alditol backbone variant of WTA synthesized, implies that the extrapolation of data obtained from *in vitro* immunological assays to the *in situ* situation in the gastrointestinal tract is certainly not trivial. To this end, the relative level of production of WTA may be altered *in situ*, as has been shown for *S. aureus* when it resides in the nasal cavity of rats [50], but also here by the observation that WTA backbone switching results in differential levels of WTA produced (Table 1). Moreover, not all *Lactobacillus* species have the capacity to produce WTA, or may alter either WTA backbone type or WTA production levels under certain conditions [2,3], which could severely impact on their signaling capacity as compared to strains that do produce this molecule. The biochemical characteristics of TAs, such as polymer length and its exact composition, including the degree of D-Ala and glycosyl substitution, may fine-tune the immune signaling effects described here. In this respect, it is intriguing that Marco and coworkers have reported the significant down-regulation of the *dltX-D* operon during passage of *L. plantarum* through the murine gastrointestinal tract [51]. Overall, the flexibility of bacteria to produce different (ratios of) TAs that have alternative biochemical characteristics (backbone and/or substitutions) under different environmental conditions is bound to impact on their host-communication capacities *in vivo*[50,51]. Moreover, inter-strain differences in the production of specific TA molecules provide a plausible explanation for the remarkable strain-specificity observed in immune assays [52,53]. Taken together, this study correlates structural differences in TAs to their functional characteristics and provides important information to improve our understanding of molecular host-microbe interactions and probiotic functionality.

## Methods

### Strains and culture conditions

*L. plantarum* WCFS1 [19] and its derivatives were cultivated in Mann-Rogusa Sharpe (MRS) broth (Merck, Darmstadt, Germany) or 2× chemically defined medium containing 1.5% glucose [54] at 37°C without agitation. When appropriate, 10 μg ml^-1^ chloramphenicol or erythromycin was added. *Escherichia coli* MC1061 [55] was used as an intermediate cloning host for the construction of the mutagenesis vectors (see below). *E. coli* was grown aerobically at 37°C in TY medium, supplemented with 10 μg ml^-1^ chloramphenicol when appropriate.

### Gene deletion mutant construction and DNA microarray analysis

*L. plantarum* gene deletion mutant construction by a double cross-over gene replacement strategy for *tagF1-F2* (*lp_0268* &*lp_0269*) and *dltX-D* (*lp_2016lp_2020*) was performed essentially as described previously [56] using the primers presented in Additional file 1: Table S3 (included in the Additional file 1: supplementary materials and methods file). RNA isolation from *L. plantarum*, subsequent cDNA synthesis and indirect labeling, as well as DNA microarray hybridizations were performed using routine procedures [51,52]. Detailed experimental procedures are reported in the Additional file 1. The custom-made 60-mer oligonucleotide array design (Agilent Biotechnologies, Amstelveen, The Netherlands) and whole-genome transcriptomics data of the WTA mutants are accessible in GEO as platform GPL9359 and dataset GSE27683.

### WTA preparation and analysis

WTAs were prepared from cell walls of the *L. plantarum* strains as described previously [8]. Briefly, the cells were grown to the stationary growth phase in 8L MRS for 20 h and harvested by centrifugation (20,000 × g for 20 min at 4°C). The cells were disrupted by ultrasonication and treated with 2% sodium dodecyl sulfate, Pronase (Roche Diagnostics, Mannheim, Germany), DNase (Wako Pure Chemical Industries, Osaka, Japan), RNase (Sigma-Aldrich, St. Louis, USA), and Pepsin (Sigma-Aldrich). After thorough washing with distilled water, cell-walls were pelleted by centrifugation (20,000 × g for 20 min at 4°C) and lyophilized. The WTAs were released from the cell walls by 10% trichloroacetic acid treatment at 4°C, collected by ethanol precipitation at 4°C, and hydrolyzed with 2N hydrochloric acid at 100°C for 3 h. Sugars and alditols in the WTA hydrolysates were identified using a high-performance liquid chromatography (HPLC, LC-10 system, Shimadzu, Kyoto, Japan) on a Shodex Asahipak NH2P-50 4E column (Showa Denko, Tokyo, Japan) with water-acetonitrile (25:75, v/v) as the eluent at 30°C [8,57]. The D-Ala content was quantified using the HPLC system on a Shodex SUGAR SC1011 column (Showa Denko) with 10 mM calcium chloride (pH 5.8) as the eluent at 80°C. Phosphoric acid, glucose, and D-Ala content in the WTAs were determined by Allen’s method [58], phenol-sulfuric acid method [59], and HPLC, respectively.

### Innate signaling and immunological assays

Buffy coats from 4 blood donors were obtained from the Sanquin blood bank (Nijmegen, The Netherlands). A written informed consent was obtained from each volunteer prior to sample collection. Monocyte isolation from human blood, generation of immature dendritic cells (iDCs), and stimulation of iDCs with LPS (final concentration of 1.0 μg/ml, positive control) or *L. plantarum* strains (1:10 ratio, all strains in duplicate from 2 independent cultures) was performed as described previously [52]. After 48h, duplicate supernatant from the DC stimulation assays were analyzed for the presence of cytokines (TNF-α, IL-12p70 and IL-10) using a cytometric bead-based immunoassay that enables multiplex measurements of soluble cytokines in the same sample [60], according to the manufacturer’s protocol (BD biosciences, Breda, The Netherlands). The limits of sensitivity for detection were: TNF-α 0.7 pg ml^-1^; IL-12p70 0.6 pg ml^-1^ and IL-10 0.13 pg ml^-1^. The flow cytometry data were analyzed using the BD FCAP software (BD biosciences, Breda, The Netherlands). For statistical analysis mixed general linear model using restricted maximum likelihood (REML) was used to determine the statistical differences within donors between cytokine produced by DCs stimulated with the constructed deletion mutants compared to the parental *L. plantarum* WCFS1 strain. A two-sided *p*-value of 0.05 or lower was considered to be significant. The statistical analysis was performed by using SAS software (version 9.1, SAS Institute Inc., Cary, NC, USA).

Human embryonic kidney (HEK)293 cells harboring pNIFTY, a NF-κB luciferase reporter construct (Invivogen, Toulouse, France) were used as the negative control in the NF-κB assays. A HEK293 cell line constitutively expressing human hTLR-2/6 was seeded at 5 × 10^5^ cells cm^-2^ in 96-well plates and incubated overnight under standard culture conditions [61]. Subsequently, these cell lines were incubated in triplicate for 6 h with 3 independent bacterial batches of *L. plantarum* WCFS1 or its derivatives (15 CFU/HEK293 cell), the positive control TLR2 ligand Pam3-Cys-SK4 (5 μg ml^-1^), or with medium alone as a negative control. NF-κB activation was measured by the Bright-glo luciferase assay (Promega Benelux BV, Leiden, The Netherlands). As anticipated, NF-κB activity significantly increased only in the cell lines transfected with hTLR-2/6. Values obtained with the *L. plantarum* strains were normalized to medium induced NF-κB activity. ANOVA multiple comparison was used to determine the statistical differences between the NF-κB induced activities stimulated with the constructed deletion mutants compared to the parental *L. plantarum* WCFS1 strain. A two-sided *p*-value < 0.05 was considered significant.

The study was approved by the Wageningen University Ethical Committee and was performed according to the principles of the Declaration of Helsinki. Buffy coats from blood donors were obtained from the Sanquin Blood bank in Nijmegen (The Netherlands). A written informed consent was obtained from all blood donors prior to sample collection.

## Competing interests

The authors declare that they have no competing interests.

## Authors’ contributions

PAB concepted the study, and drafted the manuscript. ST performed the WTA and LTA analyses and critically revised the manuscript. IIvS constructed the *tagF1-2* mutant and performed the qRT-PCR experiments. DMR performed the DNA microarray experiments and critically revised the manuscript. MM designed and performed the signalling and immune assays, and critically revised the manuscript. MW designed the DNA microarray experiments, and performed the data analysis and visualization. SO designed the WTA and LTA analyses. JMW designed the immune and signalling experiments, and critically revised the manuscript. MK conceived the study and drafted the manuscript. All authors read and approved the final manuscript.

## Supplementary Material

Additional file 1 Detailed description of mutant construction, DNA microarray analysis, and LTA and WTA chain length determination. Click here for file

Additional file 2 **Table S2.** Relative transcription levels of genes over 2-fold regulated (fdr < 0.05) in the *dltX-D* (NZ3539Cm) and *tagF1-2* (NZ3547Cm) mutants as compared to the wild-type strain. Click here for file

Additional file 3 **Figure S1.** Expression level of the *tarIJKL* operon (*lp_1816*-*1819*) compared to the median of expression under a variety of fermentation conditions. Click here for file

Additional file 4 **Figure S2.** The signaling capacity of purified WTAs was measured as NF-κB pathway activation relative to a medium only control, as measured by a luminescence reporter in HEK cell lines expressing TLR-2/6 after exposure to 0.2, 1.0, 5.0, 25 or 125 μg/ml WTA containing either a poly(Gro-P) or poly(Rbo-P) backbone (isolated from *L. plantarum* WCFS1 or the *tagF1-2* mutant, respectively). All WTAs were applied three times to the assay. Bars represent averages of these three measurements with standard deviations. Click here for file

Additional file 5 **Figure S3.** The signaling capacity of the *tagO* mutant functionally complemented with WTA was measured as NF-κB pathway activation relative to the *tagO* mutant without added WTA, as measured by a luminescence reporter in HEK cell lines expressing TLR-2/6. HEK cell lines were exposed to the *tagO* mutant (The ratios of HEK reporter to bacterial cell were 1:15) plus 0.2, 1.0, 5.0, 25 or 125 μg/ml WTA containing either a poly(Gro-P) or poly(Rbo-P) backbone (isolated from *L. plantarum* WCFS1 or the *tagF1-2* mutant, respectively). All samples were applied three times to the assay, and the bars represent averages of these three measurements with standard deviations. Click here for file

## Author details

^1^TI Food & Nutrition, Nieuwe Kanaal 9A, 6709PA, Wageningen, The Netherlands. ^2^NIZO Food Research, Kernhemseweg 2, 6718ZB, Ede, The Netherlands. ^3^Kluyver Centre for Genomics of Industrial Fermentation, Julianalaan 67, 2628 BC, Delft, The Netherlands. ^4^Department of Applied Biology and Chemistry, Faculty of Applied Bio-Science, Tokyo University of Agriculture, 1-1-1 Sakuragaoka, Setagaya-ku, Tokyo 156-8502, Japan.^5^Host-Microbe Interactomics, Wageningen University, Marijkeweg 40, 6709 PG, Wageningen, The Netherlands. ^6^Netherlands Consortium for Systems Biology, Science Park 904, 1098 XH, Amsterdam, The Netherlands.^7^Laboratory for Microbiology, Wageningen University, Dreijenplein 10, 6703 HB, Wageningen, The Netherlands.
