# Supplementary material for: Lactobacillus plantarum possesses the capability for wall teichoic acid backbone alditol switching
Source: Microb Cell Fact. 2012 Sep 11;11:123. doi: 10.1186/1475-2859-11-123 (PMC3511166; doi:10.1186/1475-2859-11-123)
Supplement: Additional file 1 — Detailed description of mutant construction, DNA microarray analysis, and LTA and WTA chain length determination. [file 1475-2859-11-123-S1.docx]

**Supplementary information for:**

***Lactobacillus plantarum* wall teichoic acid backbone switching elicits differential immune modulation**

Peter A. Bron^1,2,3^, Satoru Tomita^4^, Iris I. van Swam^1,2^, Daniela M. Remus^1,2,7^, Marjolein Meijerink^1,5,6^, Michiel Wels^1,2^, Sanae Okada^4^, Jerry M. Wells^1,5^, and Michiel Kleerebezem^1,2,6,7^

^1^TI Food & Nutrition, Nieuwe Kanaal 9A, 6709 PA Wageningen, The Netherlands

^2^NIZO food research, Kernhemseweg 2, 6718ZB Ede, The Netherlands

^3^Kluyver Centre for Genomics of Industrial Fermentation, Julianalaan 67, 2628 BC Delft, The Netherlands

^4^Department of Applied Biology and Chemistry, Faculty of Applied Bio-Science, Tokyo University of Agriculture, 1-1-1 Sakuragaoka, Setagaya-ku, Tokyo 156-8502, Japan

^5^Host-Microbe Interactomics, Wageningen University, Marijkeweg 40, 6709 PG Wageningen, The Netherlands

^6^Netherlands Consortium for Systems Biology, Science Park 904, 1098 XH Amsterdam, The Netherlands

^7^Laboratory for Microbiology, Wageningen University, Dreijenplein 10, 6703 HB Wageningen, The Netherlands

Contact: Michiel Kleerebezem, NIZO food research, Kernhemseweg 2, 6718ZB Ede, The Netherlands. E: [Michiel.Kleerebezem@nizo.nl](mailto:Michiel.Kleerebezem@nizo.nl), P: +31-(0)318-659629, F: +31-(0)318-650400

**DNA manipulations and mutant construction**

Plasmid DNA was isolated from *Escherichia coli* using Jetstar columns as recommended by the manufacturer (Genomed GmbH, Bad Oberhausen, Germany). For DNA manipulations in *E. coli*, standard procedures were used [1]. Restriction endonucleases, DNA polymerases, and T4 DNA ligase were used as specified by the manufacturers (Promega, Leiden, The Netherlands; Boehringer, Mannheim, Germany). Primers were obtained from Sigma Aldrich (Zwijndrecht, The Netherlands). Construction of the *Lactobacillus plantarum* gene deletion mutants for *tagF1-F2* (*lp_0268* & *lp_0269*) and *dltX-D* (*lp_2016*-*lp_2020*) was performed essentially as described previously [2-4] and involved a double cross-over gene replacement strategy to replace the target gene(s) by the chloramphenicol resistance cassette *lox66*-P_32_*cat*-*lox71* [2]. However, a variant of pNZ5319 designated pNZ5319TAG (Bron *et al*., unpublished data) was used as mutagenesis vector. By using this vector, a unique DNA tag is introduced in each mutant, allowing mutant-specific tracking and identification in competitive experiments (not relevant for the experiments reported here). The upstream and downstream flanking regions of the target gene(s), as well as the *lox66*-P_32_*cat*-*lox71* cassette were amplified by PCR using the corresponding primer pairs (Table S3). The resulting amplicons were used as templates in a SOEing PCR reaction [5] bridging the flanking regions and *lox66*-P_32_*cat*-*lox71* by means of complementary regions in the primers (Table S3). Subsequently, PCR products were blunt-ligated into *Swa*I-*Ecl*136II digested pNZ5319TAG, which can replicate in the intermediate cloning host *E. coli*, but can be used as a suicide integration vector in *L. plantarum* [2]. The integrity of the cloned amplicons was confirmed by sequence analysis (BaseClear, Leiden, The Netherlands). The mutagenesis plasmids were transformed to *L. plantarum* WCFS1 [6] and colonies displaying a chloramphenicol-resistant and erythromycin-sensitive phenotype, representing candidate homologous recombination double-cross events, were selected [2]. The anticipated *cat*-replacement genotype was confirmed by PCR using primers flanking the sites of recombination. A single colony of the *tagF1-F2* and *dltX*-*D* transformations displaying the anticipated genotype and phenotype was selected for further analysis and designated NZ3547Cm and NZ3539Cm, respectively (Table S3).

**DNA microarrays**

RNA isolation from *L. plantarum*, subsequent cDNA synthesis and indirect labeling, as well as DNA microarray hybridizations were performed using routine procedures [3, 7]. *L. plantarum* WCFS1 and its derivatives were grown in 2× CDM medium containing 1.5% glucose [8] until an OD_600_ of 1.0 was reached, followed by quenching [9] and RNA isolation. Subsequently, 5 µg of RNA was used for cDNA synthesis and indirect labeling with cyanine 5 or cyanine 3 [3, 7]. The microarray hybridization scheme consisted of 3 arrays and the following cDNA samples per array were hybridized with cyanine 3 and cyanine 5, respectively: NZ3547Cm and WCFS1, WCFS1 and NZ3539Cm, NZ3539Cm and NZ3547Cm. A two-color microarray-based gene expression analysis was performed on a custom-made 60-mer oligonucleotide array (Agilent Biotechnologies, submitted in GEO under platform GPL9359) to determine genome-wide differential gene transcription levels. Co-hybridization of Cy5- and Cy3-labeled cDNA probes was performed on these oligonucleotide arrays at 42°C for 16 h in Slidehyb#1 (Ambion, Austin, USA). Subsequently, the slides were washed twice in 1× SSC containing 0.1% sodium dodecyl sulfate and twice in 1× SSC before they were scanned. Slides were scanned with a ScanArray Express 4000 scanner (Perkin Elmer, Wellesley, USA), and image analysis and processing were performed using the ImaGene Version 7.5 software (BioDiscovery Inc., Marina Del Rey, USA). The microarrays were scanned at different intensities and for each of the microarrays the best scan was selected on the basis of signal distribution (combination of a low number of saturated spots and a low number of low signal spots). The data were normalized using Lowess normalization as available in MicroPrep [10]. The data were corrected for inter-slide differences on the basis of total signal intensity per slide using Postprep [10]. The median intensity of the different probes per gene was selected as the gene expression intensity. CyberT was used to compare the different transcriptomes, taking into account the duplicates (dye swaps) of each of the conditions [11]. This analysis resulted in a gene expression ratio and false discovery rate (FDR) for each gene. Genes with FDR values <0.05 were considered to be statistically significant. All microarray data is MIAME compliant and are available in the GEO database (GSE27683)***.*** For data visualization (Fig. 4) the simpheny software package was used (www. genomatica.com), using the *L. plantarum* specific model [12].

**WTA and LTA backbone polymer chain length determination**

WTA was isolated as described in the main manuscript. LTAs of *Lactobacillus plantarum* were prepared by n-butanol extraction, followed by hydrophobic interaction chromatography as described previously [13]. Subsequently, LTA was fractionated by an AKTA fast performance liquid chromatography system (GE Healthcare) on a DEAE-Sepharose Fast Flow column (5.0 × 5 cm, GE Healthcare) with a linear gradient of 0−1.0 M sodium chloride in 50 mM sodium acetate buffer (pH 4.7). The fractions containing organic phosphate were pooled, dialyzed against water, and collected as purified LTA after lyophilization. Proton NMR spectra of purified LTAs were recorded on an AVANCE 500 MHz NMR spectrometer (Bruker) in deuterium oxide at 60°C with a proton frequency of 500.13 MHz. Sodium 3-(trimethylsilyl)propionic-2,2,3,3-d_4_ acid was used as an internal reference of chemical sift (δ_H_, 0.00 ppm). Average chain length of poly(Gro-P) backbone of LTAs was obtained from calculation of the integral ratio of the backbone and the methyl residues in fatty acids (Table S1).

For WTA chain length determination high performance size exclusion chromatography was performed on an Ultimate 3000 system (Dionex) equipped with a set of three TSK-GEL columns (TSK-GEL super AW2500, AW3000, and AW4000) with 0.2 M sodium nitrate at 55°C. Molecular size of WTAs was estimated by performing the same analysis using a range of pectin standards (10−100 kDa) as a calibration curve (Table S1).

**Table S1:** Chain length of WTA and LTA in *L. plantarum* WCFS1 and the *tagO*, *dltX-D*, and *tagF1-2* gene deletion mutants. n.d. is not detectable.

| Chain length: | WCFS1: | Δ*tagO*: | Δ*dltX−D*: | Δ*tagF1−2*: |
| --- | --- | --- | --- | --- |
| WTA (kDa) | 30.9 | n.d. | 30.0 | 23.4 |
| LTA (units) | 33.6 | 32.9 | 34.7 | 26.1 |

**Table S3:** Primers used for the construction of gene deletion mutants in *L. plantarum* WCFS1. Primerpairs is153-154 and is93-142 were used for amplification of the upstream regions of NZ3547Cm and NZ3539Cm, whereas primerpairs is161-162 and is143-96 were used for generation of the downstream amplicons, respectively.

| Primer name: | Primer sequence*^a^* (5’ to 3’): | Target region: |
| --- | --- | --- |
| is128 tag-lox66-F3 | aaatctaccgttcgtataatgtatg | *lox66-cat-lox71* |
| is129 tag-lox71-R3 | ctcatgcccgggctgtaaccg | *lox66-cat-lox71* |
|  | **NZ3547Cm (*tagF1&F2::cat*) / (*lp_0268&0269::cat*)** |  |
| is153 lp0267F | Gcaaatgatccgcgatccgc | *tagF1&F2 (lp_0268-0269)* |
| is154 lp0267R | gcatacattatacgaacggtagattttgccattttattgaattccccg | *tagF1&F2 (lp_0268-0269)* |
| is161 lp0270F | cggttacagcccgggcatgagactagagcaattgtttgagctgg | *tagF1&F2 (lp_0268-0269)* |
| is162 lp0270R | cgtgctggcagttagtgtgg | *tagF1&F2 (lp_0268-0269)* |
|  | **NZ3539Cm (*dltX-D::cat*) / (*lp_2016-2020::cat*)** |  |
| is93 lp2015F | atttttcgcgttcgtgaccg | *dltX-D (lp2016-2020)* |
| is142 lp2015R | gcatacattatacgaacggtagattttaacctactaaaaagtacaggcc | *dltX-D (lp2016-2020)* |
| is143 lp2021F | cggttacagcccgggcatgaggcaaacatgccatcacctcc | *dltX-D (lp2016-2020)* |
| is96 lp2021R | cgtaaagtcaatcagacgacg | *dltX-D (lp2016-2020)* |

*^a^* Underlined nucleotides indicate overlapping ends with is128 tag-lox66-F3/ is129 tag-lox71-R3

**References**

1. Sambrook J, Fritsch EF, Maniatis T: **Molecular cloning: a laboratory manual** *Cold Spring Harbor, New York* 1989, **2nd Ed.**

2. Lambert JM, Bongers RS, Kleerebezem M: **Cre-*lox*-based system for multiple gene deletions and selectable-marker removal in *Lactobacillus plantarum***. *Appl Environ Microbiol* 2007, **73**(4):1126-1135.

3. Meijerink M, van Hemert S, Taverne N, Wels M, de Vos P, Bron PA, Savelkoul HF, van Bilsen J, Kleerebezem M, Wells JM: **Identification of genetic loci in *Lactobacillus plantarum* that modulate the immune response of dendritic cells using comparative genome hybridization**. *PLoS One* 2010, **5**(5):e10632.

4. van Hemert S, Meijerink M, Molenaar D, Bron PA, de Vos P, Kleerebezem M, Wells JM, Marco ML: **Identification of *Lactobacillus plantarum* genes modulating the cytokine response of human peripheral blood mononuclear cells**. *BMC Microbiol* 2010, **10**:293.

5. Horton RM: **PCR-mediated recombination and mutagenesis. SOEing together tailor-made genes**. *Mol Biotechnol* 1995, **3**(2):93-99.

6. Ferain T, Garmyn D, Bernard N, Hols P, Delcour J: ***Lactobacillus plantarum ldhL* gene: overexpression and deletion**. *J Bacteriol* 1994, **176**(3):596-601.

7. Marco ML, Peters TH, Bongers RS, Molenaar D, van Hemert S, Sonnenburg JL, Gordon JI, Kleerebezem M: **Lifestyle of *Lactobacillus plantarum* in the mouse caecum**. *Environ Microbiol* 2009, **11**(10):2747-2757.

8. Teusink B, van Enckevort FH, Francke C, Wiersma A, Wegkamp A, Smid EJ, Siezen RJ: ***In silico* reconstruction of the metabolic pathways of *Lactobacillus plantarum*: comparing predictions of nutrient requirements with those from growth experiments**. *Appl Environ Microbiol* 2005, **71**(11):7253-7262.

9. Pieterse B, Jellema RH, van der Werf MJ: **Quenching of microbial samples for increased reliability of microarray data**. *J Microbiol Methods* 2006, **64**(2):207-216.

10. van Hijum SA, Garcia de la Nava J, Trelles O, Kok J, Kuipers OP: **MicroPreP: a cDNA microarray data pre-processing framework**. *Appl Bioinformatics* 2003, **2**(4):241-244.

11. Baldi P, Long AD: **A Bayesian framework for the analysis of microarray expression data: regularized t -test and statistical inferences of gene changes.** *Bioinformatics* 2001, **17**:509–519.

12. Teusink B, Wiersma A, Molenaar D, Francke C, de Vos WM, Siezen RJ, Smid EJ: **Analysis of growth of *Lactobacillus plantarum* WCFS1 on a complex medium using a genome-scale metabolic model**. *J Biol Chem* 2006, **281**(52):40041-40048.

13. Morath S, Geyer A, Hartung T: **Structure-function relationship of cytokine induction by lipoteichoic acid from *Staphylococcus aureus***. *J Exp Med* 2001, **193**(3):393-397.
