# Supplementary figures and images for: Lactobacillus plantarum possesses the capability for wall teichoic acid backbone alditol switching
Source: Microb Cell Fact. 2012 Sep 11;11:123. doi: 10.1186/1475-2859-11-123 (PMC3511166; doi:10.1186/1475-2859-11-123)

## Slide 1
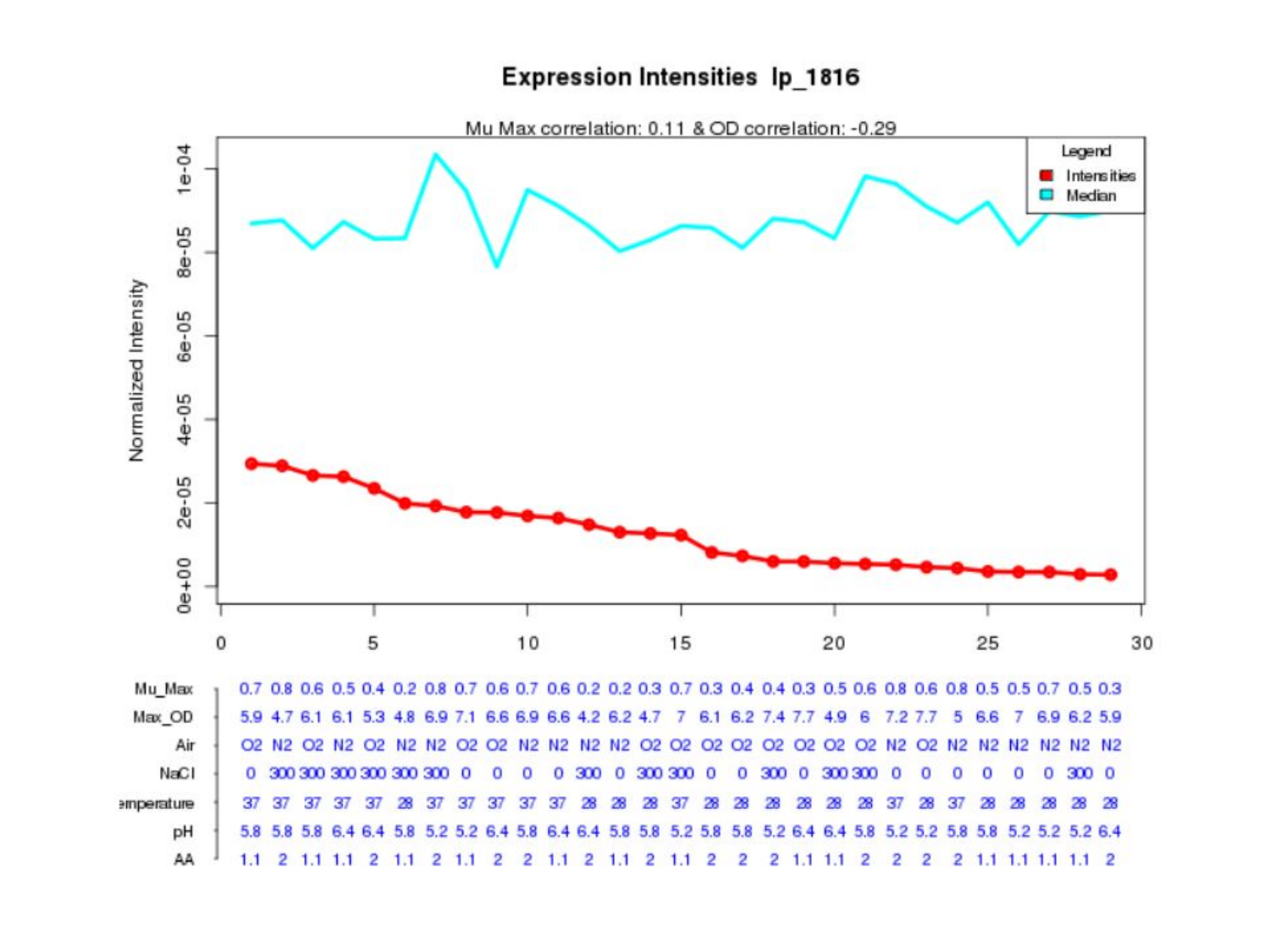

## Slide 2
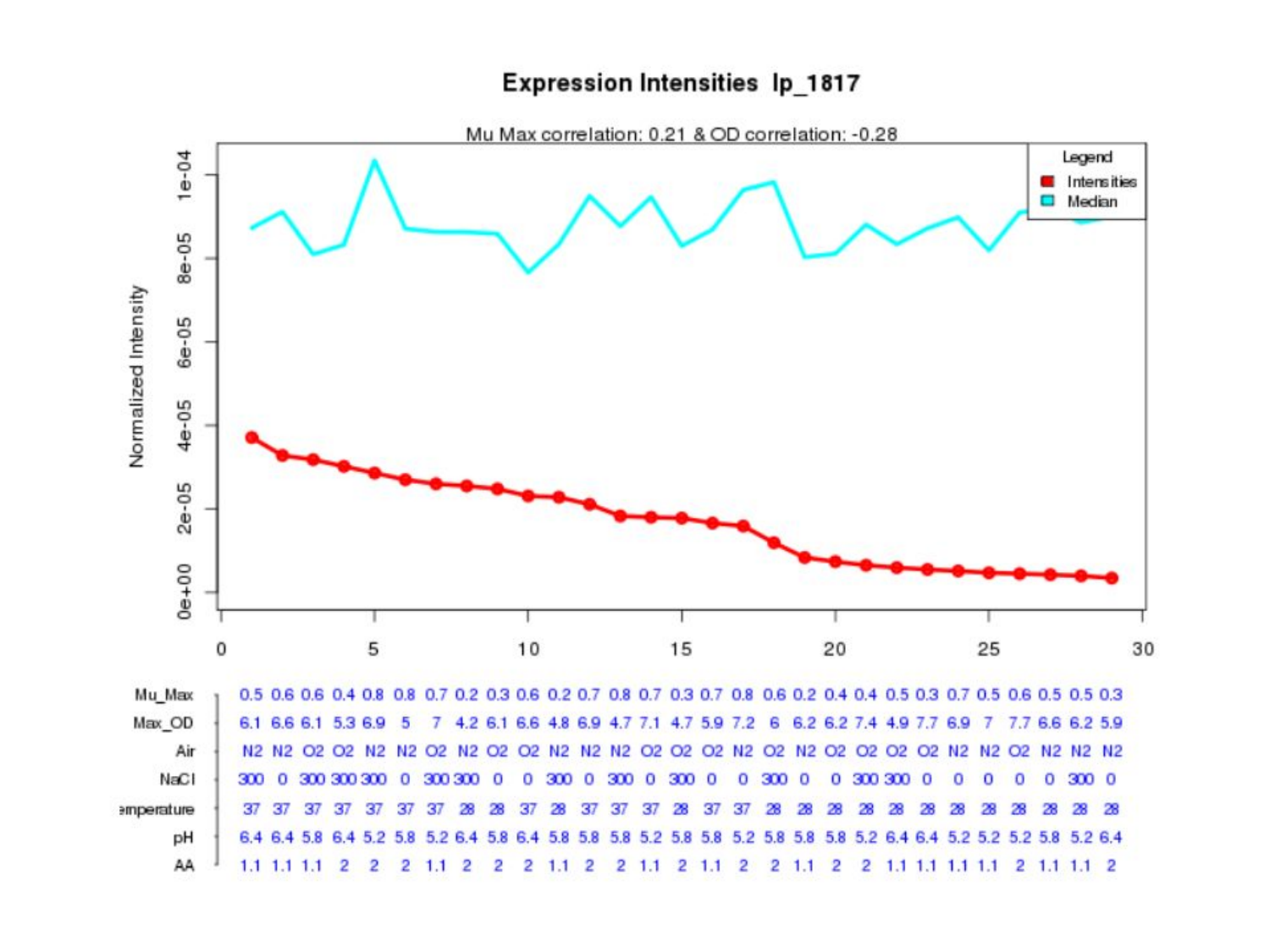

## Slide 3
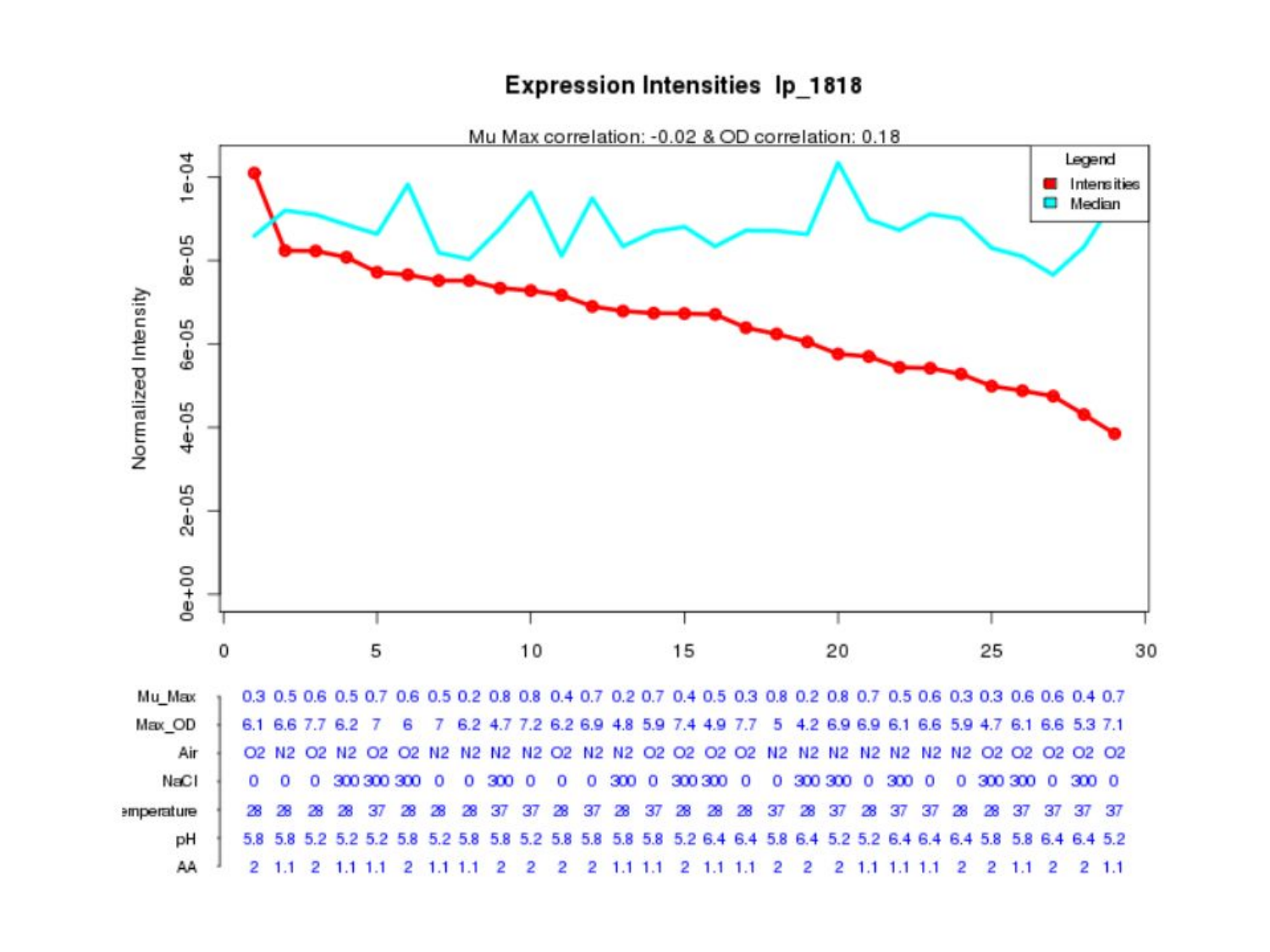

## Slide 4
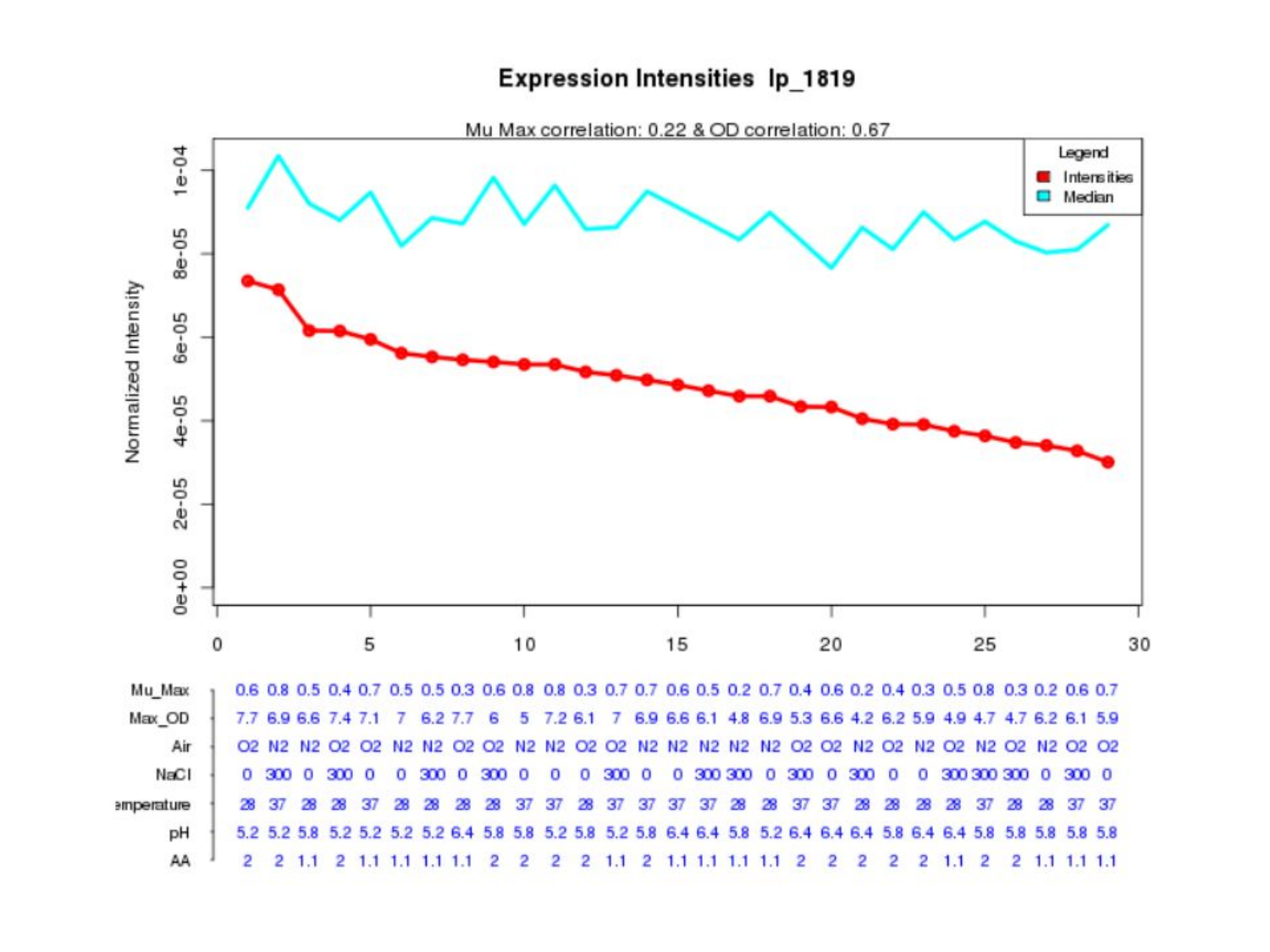

Supplement: Additional file 3 — Figure S1. Expression level of the tarIJKL operon (lp_1816-1819) compared to the median of expression under a variety of fermentation conditions. [file 1475-2859-11-123-S3.pptx]

## Slide 1
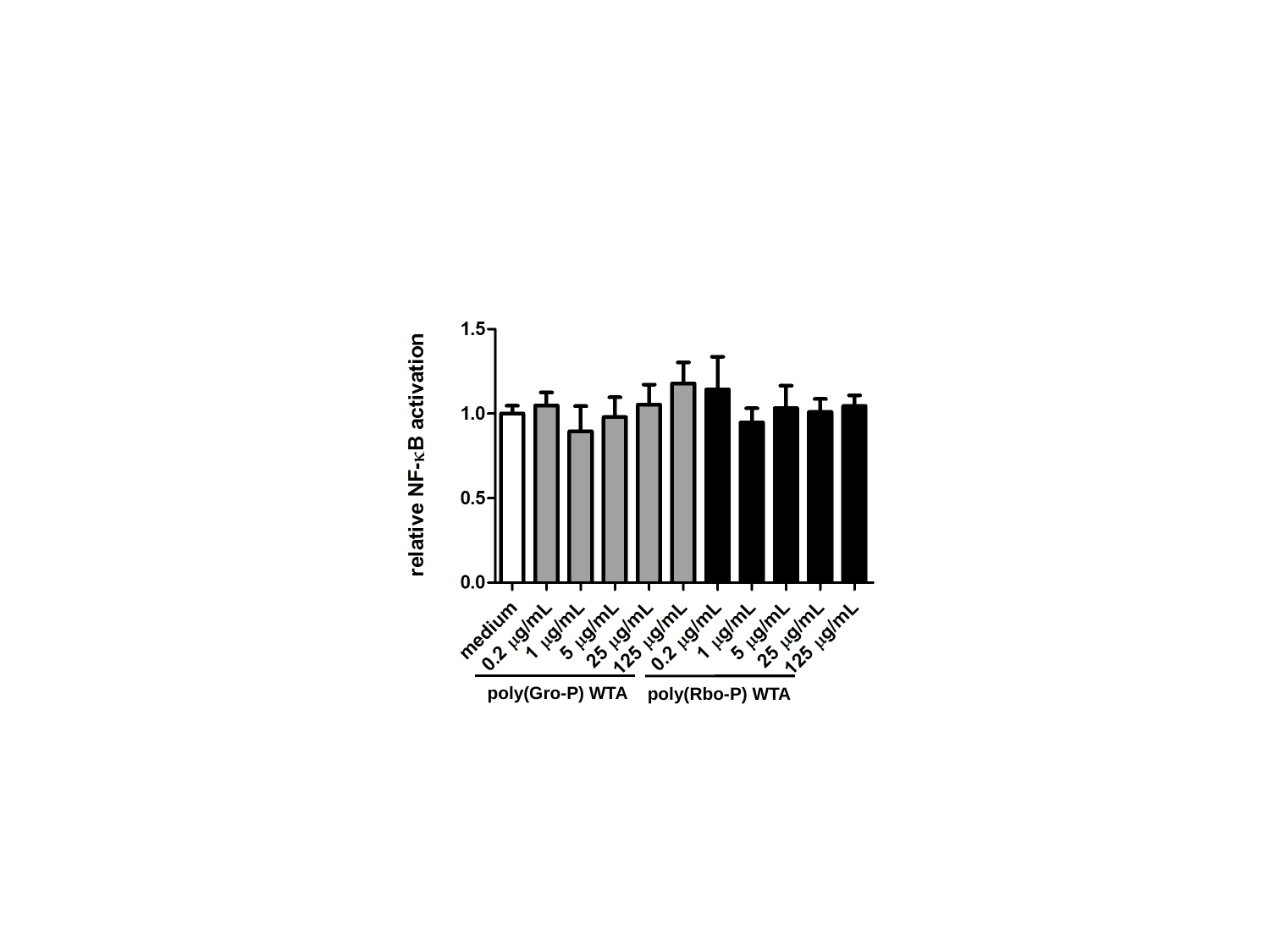

poly(Gro-P) WTA
poly(Rbo-P) WTA

Supplement: Additional file 4 — Figure S2. The signaling capacity of purified WTAs was measured as NF-κB pathway activation relative to a medium only control, as measured by a luminescence reporter in HEK cell lines expressing TLR-2/6 after exposure to 0.2, 1.0, 5.0, 25 or 125 μg/ml WTA containing either a poly(Gro-P) or poly(Rbo-P) backbone (isolated from L. plantarum WCFS1 or the tagF1-2 mutant, respectively). All WTAs were applied three times to the assay. Bars represent averages of these three measurements with standard deviations. [file 1475-2859-11-123-S4.pptx]

## Slide 1
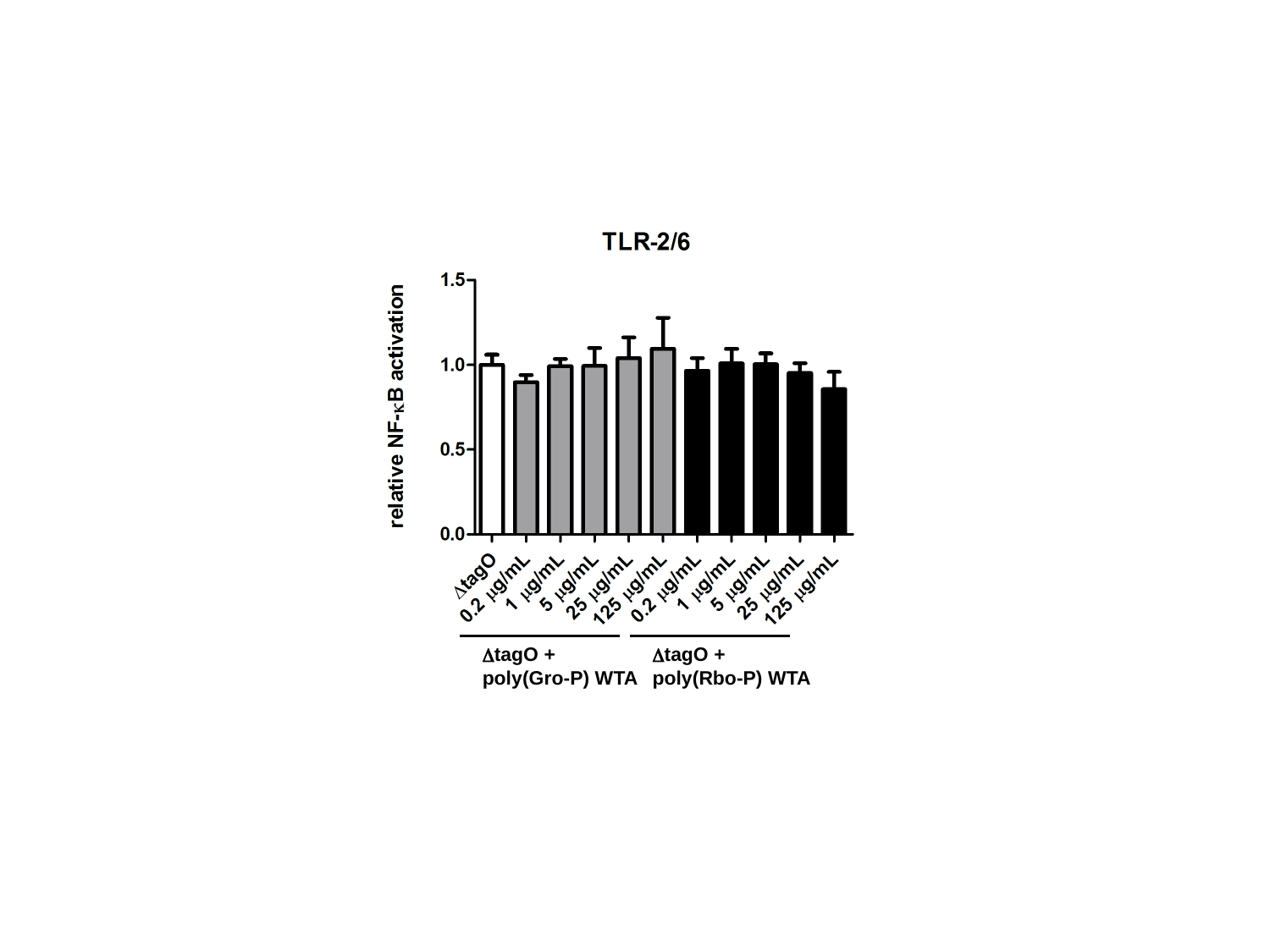

DtagO +
poly(Gro-P) WTA
DtagO +
poly(Rbo-P) WTA

Supplement: Additional file 5 — Figure S3. The signaling capacity of the tagO mutant functionally complemented with WTA was measured as NF-κB pathway activation relative to the tagO mutant without added WTA, as measured by a luminescence reporter in HEK cell lines expressing TLR-2/6. HEK cell lines were exposed to the tagO mutant (The ratios of HEK reporter to bacterial cell were 1:15) plus 0.2, 1.0, 5.0, 25 or 125 μg/ml WTA containing either a poly(Gro-P) or poly(Rbo-P) backbone (isolated from L. plantarum WCFS1 or the tagF1-2 mutant, respectively). All samples were applied three times to the assay, and the bars represent averages of these three measurements with standard deviations. [file 1475-2859-11-123-S5.pptx]
